# Wnt/β-catenin signaling pathway inhibits the proliferation and apoptosis of U87 glioma cells via different mechanisms

**DOI:** 10.1371/journal.pone.0181346

**Published:** 2017-08-24

**Authors:** Liyang Gao, Bing Chen, Jinhong Li, Fan Yang, Xuecheng Cen, Zhuangbing Liao, Xiao’ao Long

**Affiliations:** 1 School of Life Science, Ningxia University, Yinchuan, China; 2 Stem Cell Research and Cellular Therapy Center, Affiliated Hosptial of Guangdong Medical University, Zhanjiang, China; 3 Department of Neurosurgery, Affiliated Hospital of Guangdong Medical University, Zhanjiang, China; University of Navarra, SPAIN

## Abstract

The Wnt signaling pathway is necessary for the development of the central nervous system and is associated with tumorigenesis in various cancers. However, the mechanism of the Wnt signaling pathway in glioma cells has yet to be elucidated. Small-molecule Wnt modulators such as ICG-001 and AZD2858 were used to inhibit and stimulate the Wnt/β-catenin signaling pathway. Techniques including cell proliferation assay, colony formation assay, Matrigel cell invasion assay, cell cycle assay and Genechip microarray were used. Gene Ontology Enrichment Analysis and Gene Set Enrichment Analysis have enriched many biological processes and signaling pathways. Both the inhibiting and stimulating Wnt/β-catenin signaling pathways could influence the cell cycle, moreover, reduce the proliferation and survival of U87 glioma cells. However, Affymetrix expression microarray indicated that biological processes and networks of signaling pathways between stimulating and inhibiting the Wnt/β-catenin signaling pathway largely differ. We propose that Wnt/β-catenin signaling pathway might prove to be a valuable therapeutic target for glioma.

## Introduction

The Wnt/β-catenin pathway is a highly conserved pathway that contains Wnt proteins, Frizzled receptor families, low-density lipoprotein-related protein receptors, cytoplasmic proteins, such as Dishevelled, Axin, glycogensynthase kinase 3 (GSK3) glycoproteins, APC, and transcription factors such as β-catenin, T-cell factor/lymphoid enhancer factor (TCF/LEF) [1,2]. This pathway is one of the well-established signaling pathways during tumorigenesis and plays an important role in the development of the central nervous system. In addition, it triggers the neural differentiation of embryonic stem cells in vitro. The Wnt/β-catenin also helps protect neural connections throughout life [3]. However, mutations of component in the Wnt pathway were found to be associated with multiple cancers. This finding suggests that function loss of certain components in Wnt cascades triggers cancer development [4]. Studies have recently suggested that the Wnt/β-catenin signaling pathway can potentially regulate the growth of gliomas. Reis et al.[5] indicated that sustained endothelial Wnt/β-catenin signaling could cause diminished angiogenesis in murine glioma models. Duan et al. [6] showed that Wnt/ pathway could regulate tumor progression, thereby manipulating the molecules of the Wnt pathway, which could suppress the growth of malignant gliomas [7–11]. Nevertheless, the roles of the Wnt/β-catenin signaling pathway in malignant gliomas are poorly studied compared with that in other cancers. Therefore, the present study was designed to evaluate the effect of the Wnt/β-catenin signaling pathway on the behavior of glioma and explore the genome-wide gene profiles regulated by this pathway. Hopefully, this study may provide sufficient information for pharmacological purposes and further provide another treatment for gliomas.

## Material and methods

### Cell culture and drug treatment

U87 glioma cells were cultured in high-glucose DMEM (C11995500BT, Gibco, USA) supplemented with 10% fetal calf serum (10099–141, Gibco, Australia) and 1× penicillin–streptomycin solution. When the cells reach confluence, 0.25% Trypsin (Gibco, USA) was used to dissociated cells before washing by 1× sterile phosphate-buffered saline (PBS; Gibco, CarIsbad, CA, USA).

### RNA preparation

Total RNA was extracted using the E.Z.N.A^TM^ Total RNA Kit I (OMEGA Bio-tek, USA). The purity of RNA was determined by measuring the OD260/280 ratio (1.7–2.1). The integrity of RNA was assessed by identifying the 28S and 18S rRNA bands after electrophoresis. Reverse transcription of 1 μg RNA to cDNA was performed using PrimeScript^TM^ RT Master Mix (RR036A, TaKaRa, Japan) following the manufacturer’s instructions.

### Affymetrix microarrays

Three groups of U87 cells were comparatively investigated by hybridization: non-treated group (control), Wnt inhibitor-treated group, Wnt stimulator-treated group. Two biological replicate samples were assayed by whole transcriptome expression profiling (Affymetrix, Santa Clara, USA). The GO analysis was examined according to Gene Ontology project (http://www.geneontology.org). The GSEA analysis was examined according to MSigBD V5.1. Pathway analysis is a functional analysis mapping genes to KEGG pathways.

### Protein expression analysis

For immunocytochemistry, the samples were washed with PBS and then fixed with 4% paraformaldehyde (PFA; Beyotime, China), followed by permeabilization using PBS/1% triton X-100 (Sigma–Aldrich) for 10 min. All samples were washed with PBS and then stained with primary and secondary antibodies. For Western blot analysis, cells were dissociated from a petri dish and then lysed using Protein Lysate Kit (Beyotime, China). Protein concentration was analyzed using the BCA Protein Assay Kit (Beyotime, China). Several primary antibodies were used, including a Monoclonal Anti-POU5F1 (OCT4) antibody(1:1000; Sigma–Aldrich, Saint Louis, MO, USA), anti-NESTIN (1:50; Beyotime, China), neuronal class III β-tubulin (1:250, Beyotime, China), anti- glial fibrillary acidic protein (GFAP) (MXB, China), anti-β-catenin (1:50–500; Santa Cruz Biotechnology, Santa Cruz, CA, USA), anti-Ki67 (MXB, China), anti-NANOG (1:1000, Sino Biological Inc., China), anti-SOX2 (1:1000, Sino Biological Inc., China). Among the secondary antibodies used were Alexa Fluor 488 goat-anti mouse IgG2α (1:1000; Life Technologies), Alexa Fluor 488 goat-anti mouse IgG (H+L) (1:1000; Life Technologies, USA), and FITC-conjugated AffiniPure Goat Anti-Rabbit IgG (H+L) (1:50–200; Jackson ImmunoResearch, US). 4, 6 Diamidino-2-phenylindole (DAPI; Shengong Tech, China) and propidium iodide (PI; Beyotime, China) were used for nucleus staining. Cells stained without primary or secondary antibodies were used as technical controls. The Leica DMI 3000B microscope was used to capture the fluorescent images of cells.

### Cell proliferation and single colony formation assay

Cells were cultured in a 96-well plate (Corning, USA) at a density of 2000 cells/100 μL for 2 d, and 10 μL of CCK-8 (Beyotime, China) was added to assess cell viability. Viable cells were quantified by measuring absorbance at 450 nm with Multiskan MK3 (Thermo Electron Corp., USA). Single colony formation assay was used to identify the ability of propagation in vitro. A total of 500 cells were plated in 6 mm petri dish and cultured for 1 wk. The clones were fixed with methanol and stained with methylrosanilnium chloride solution (Leagene, China) for 10 min and then washed completely. Stained clones that contained more than 50 cells/clone were counted and then calculated using the formula cloning efficiency = (clone number/total cell number) × 100%.

### Cell cycle analysis

Cells in the log phase of growth were collected and fixed with 70% ice-cold ethanol for 24 h at 4°C. The fixed samples were stained using the Cell Cycle and Apoptosis Analysis Kit (Beyotime, China) according to the manufacturer’s instructions. The staining solution contains buffer, PI, and RNase A. The stained samples were analyzed using BD FACSCanto II (BD, USA).

### Construction of EGFP expression in U87 cells

U87 cells were plated in 6-well plates at a density of 5 × 10^5^ cells/well a day before the lentiviral transduction. When the cells reached 40%–60% confluence, recombinant lentiviruses (GenePharma, China) were added into a complete medium with 5 μg /mL polybrene (GenePharma, China). The transduction medium was replaced with a fresh complete medium 24 h after infection. Cells were then selected using Hyclone Puromycin, and the Puromycin-resistant EGFP-U87 cells were stored for further use.

### 3D tumor spheroid invasion assays

EGFP-U87 cells were collected after 90% confluence was reached, and the cell suspension was transferred to the cover of the petri dish at a density of 5000 cells/drop. The petri dishes were then transferred gently into the incubator continuously for 2 d. The aggregates were collected with a Pasteur pipette and then mildly washed with a fresh cold medium. The ice-cold aggregate suspension was gently mixed with a cold Matrigel basement membrane matrix (Corning, USA), and the mixture was placed in a 12-well-plate. The plates were transferred to the incubator 30 min before the solid gel was covered with a warm complete medium. The shape and size of the spheroids were checked daily under a fluorescent microscope (Leica DMI 3000B).

## Results

### Specific markers expressed in U87 cells

**Fig 1:**
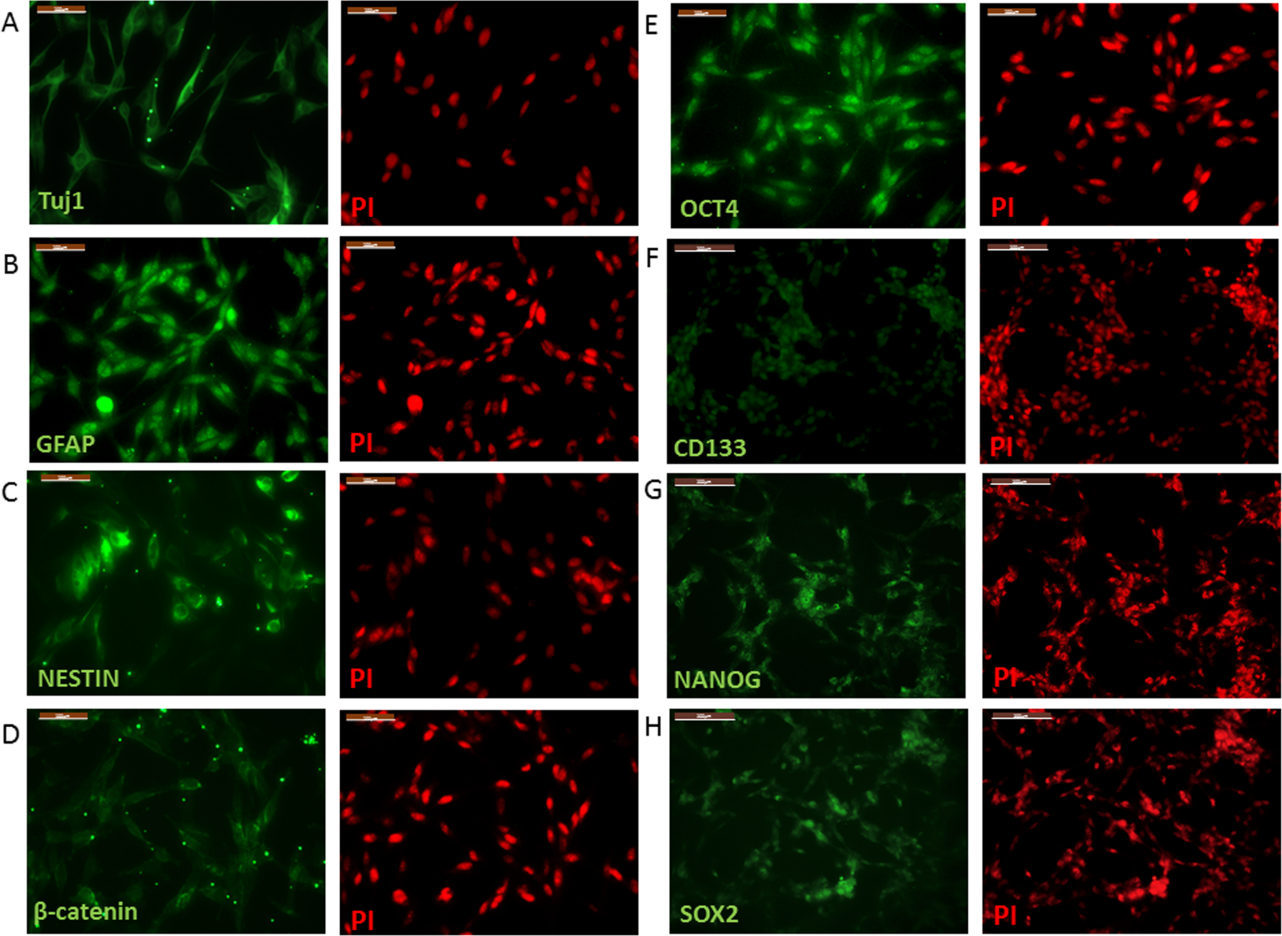
Immunofluorescence images of cultured U87 glioma cells. neural marker Tuj1 (A), neural progenitor cells marker NESTIN (B, green), glial marker GFAP (C, green), stem cell marker OCT4 (D, green), NANOG (G, green), SOX2 (H, green). The glioma stem cell marker CD133 is showed in (F, green). β-catenin (D, green) can be monitored in U87 glioma cells. Nuclei indicated by PI (red) in all images. Bars: A-E, 100um; F-H, 200um.

To examine the characteristics of U87 cells, specific markers were examined, including NESTIN, TuJ 1 neuron-specific class III β-tubulin, GFAP, CD133, OCT4, NANOG, and SOX2. These markers have been confirmed to be associated with glioma and cancer cells with stem-like properties. Immunocytochemistry (ICC) results indicated that the neural progenitor marker NESTIN, post-mitotic neuron marker TuJ 1, and glial cell marker GFAP were expressed in U87 glioma cells (Fig 1). Stem cell markers OCT4, NANOG and SOX2 were also expressed in U87 cells (Fig 1). The pentaspan transmembrane glycoprotein CD133 antigen, which is used as a specific marker for GSCs, was also expressed in U87 cells (Fig 1).

### Stimulating and inhibiting Wnt/β-catenin signaling pathway in U87 cells

**Fig 2:**
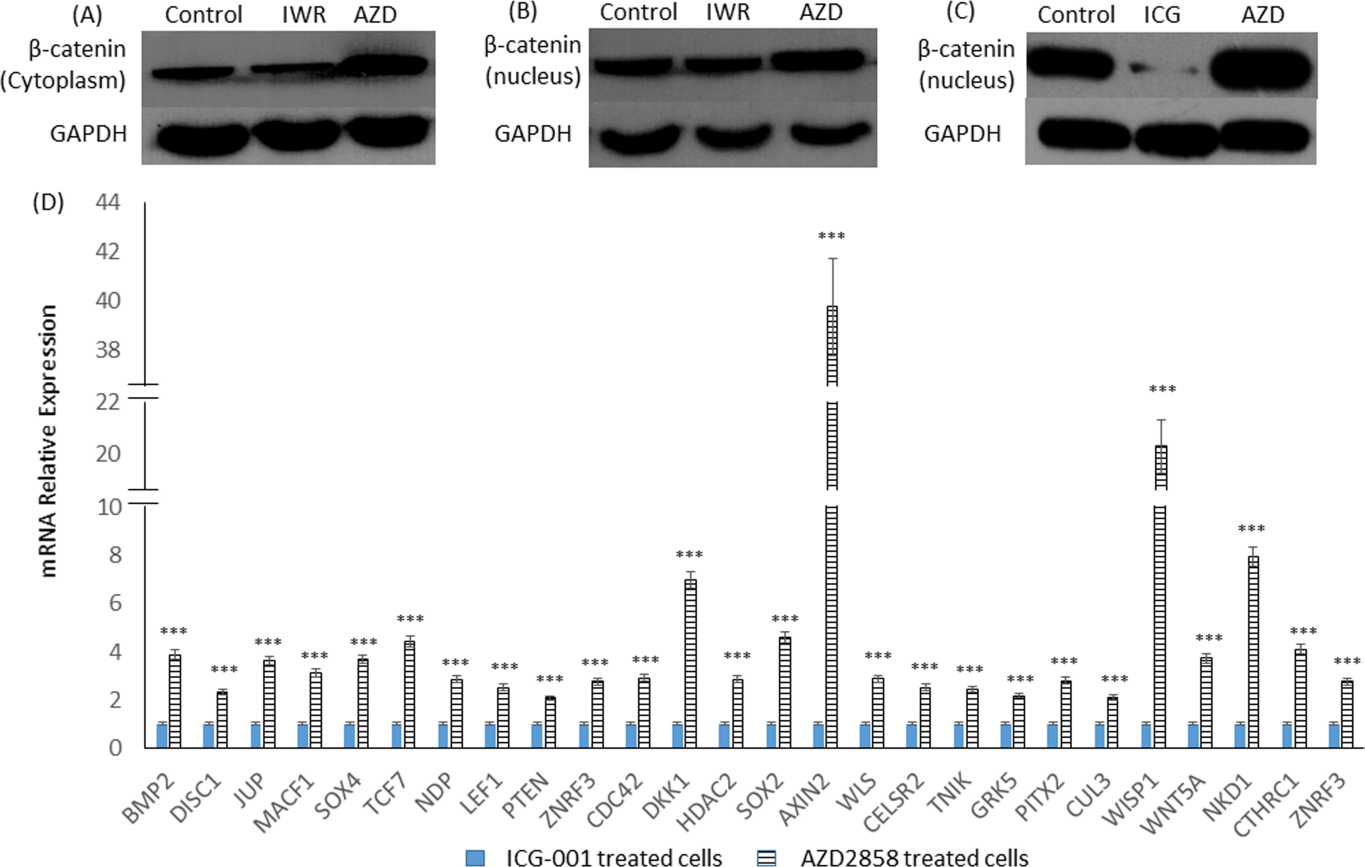
Stimulating and inhibiting Wnt/β-catenin in U87 glioma cells. (A-C) β-catenin localized to cytoplasm and nucleus under the treatment of IWR-1-endo (IWR), ICG-001 (ICG) and AZD2858 (AZD). (D) Stimulating Wnt/β-catenin signaling pathway promoted expression of Wnt/beta-catenin signaling pathway target genes, compared with inhibiting Wnt/β-catenin signaling pathway. *** means *p* < 0.001.

To inhibit the Wnt/β-catenin signaling pathway, 2 small molecules IWR-1-endo and ICG-001 were used. IWR-1-endo can stabilize the Axin destruction complex, and ICG is able to antagonize Wnt/β-catenin/TCF-mediated transcription. By contrast, AZD2858, which inhibits GSK-3, was used to activate theβ-catenin/TCF-mediated transcription. The proteins were extracted from the cytoplasm and nucleus of ICG-001, IWR-1-endo, and AZD2858-treated cells. The expression of β-catenin in the cytoplasm and nucleus of cells were detected by Western blot analysis, respectively. The results showed that IWR-1-endo could reduce the accumulation of β-catenin in the cytoplasm; however, it failed to reduce the accumulation of β-catenin in the nucleus (Fig 2). However, ICG-001 could significantly reduce the accumulation of β-catenin in the nucleus (Fig 2). By contrast, AZD2858 could enhance the accumulation of β-catenin in both the cytoplasm and the nucleus (Fig 2).

To determine the expression of the Wnt/β-catenin signaling target genes in ICG-001- and AZD2858-treated cells, Affymetrix GeneChip was used. The result indicated that the expression of the Wnt pathway target genes was significantly increased after recruiting β-catenin in the nucleus (Fig 2). This result suggested that AZD2858 could stimulate this signaling pathway, and it exerted effect completely opposite to those of ICG-001.

### Effects of Wnt/β-catenin signaling on proliferation, colony formation, and invasion of U87 glioma cells

**Fig 3:**
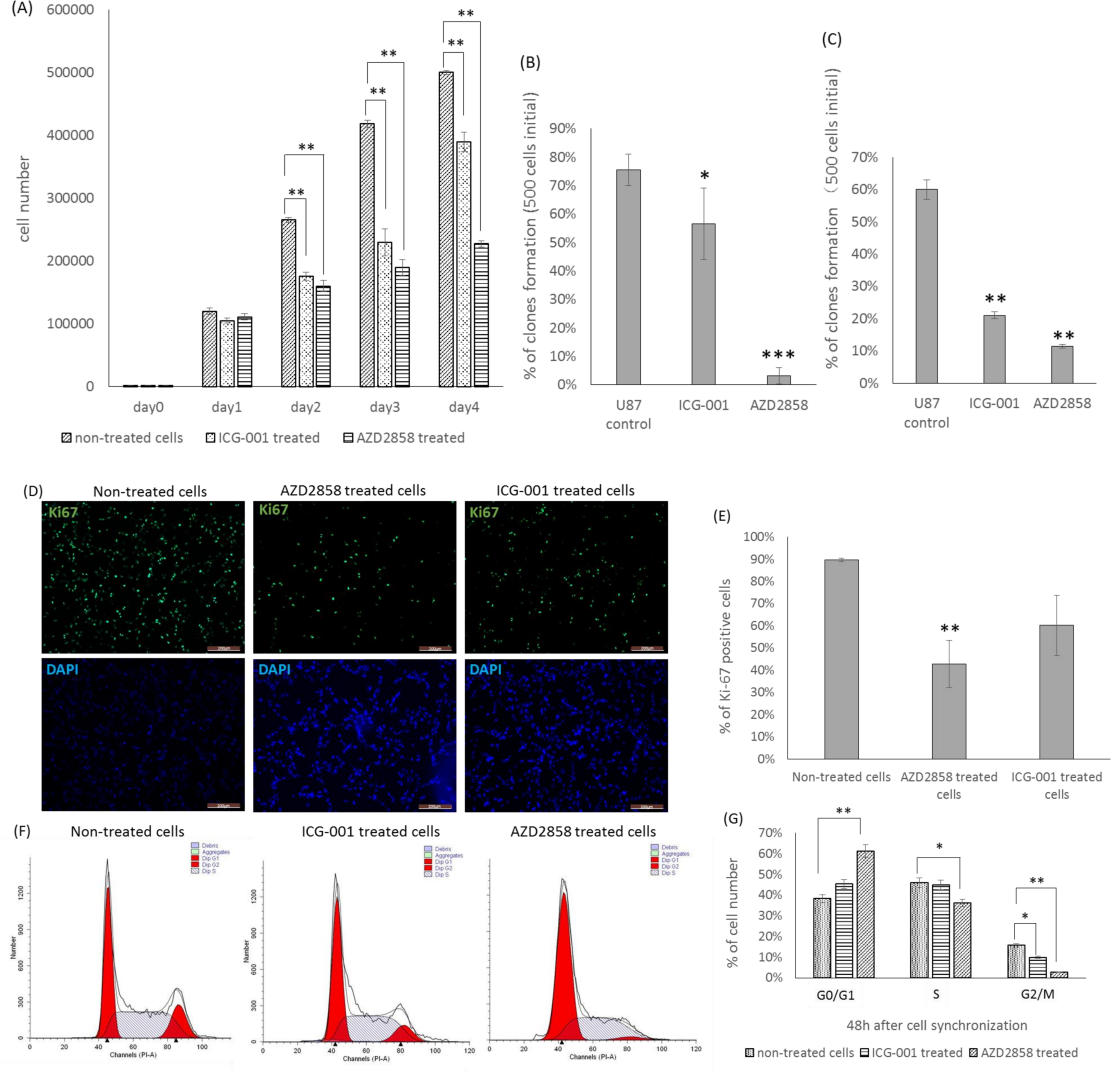
Effects of ICG-001 and AZD2858 on cell proliferation, colony formation and cell cycle of U87 glioma cells. (A) Cell viability for each media condition. (B) Percentage of single colony forming under different media condition. (C) Single colony forming of ICG-001 treated cells, AZD2858 treated cells and control cells under the same media condition. (D) Ki67 expression (green) in each groups. (E) Percentage of Ki67 positive cells. (F) Cell cycle of AZD2858 treated cells, ICG-001 treated cells and control. (G) Percentage of cells in G_0_/G_1_ phase, S phase and G2/M phase, 48 hour after cell synchronization. * means *p* < 0.05; ** means 0.001<*p* < 0.01; *** means *p* < 0.001.

To determine the cell proliferation of AZD2858- and ICG-001-treated cells, CCK-8 assay was conducted. The CCK-8 result showed that cell proliferation decreased in both groups (Fig 3). However, the decrease was greater in the AZD2858-treated cells than in the ICG-001 treated cells (Fig 3).

To identify the propagation ability of cells, single colony formation assay was performed. A total of 500 cells were plated into each petri dish, and AZD2858 and ICG-001 were added into the medium. This assay showed that less single clones were formed in the dishes containing AZD2858 and ICG-001 than control dishes (Fig 3). Moreover, to evaluate the constitutive effects of AZD2858 and ICG-001 on U87 cells, we treated U87 cells with AZD2858 and ICG-001 before the colony formation assay. For this assay, 500 AZD2858 or ICG001-treated cells were plated into each petri dish containing a drug-free, complete medium. The result indicated that AZD2858- and ICG-001-treated cells had greater difficulty forming a single cell-derived clone compared with the non-treated control cells (Fig 3). This result suggested that impairment to cell propagation could continue even if the treatment was discontinued. The effects of ICG-001 and AZD2858 treatment could last in U87 cells for a period of time.

To identify the cell proliferation of AZD2858- and ICG-001-treated cells, the mitotic marker Ki67 was used. ICC images showed that the Ki67 was monitored in all 3 groups (Fig 3). ImageJ analysis was also used to determine the percentage of Ki67 positive cells. Results indicated that the AZD2858-treated cells have a lower percentage of Ki67-positive cells than the control and ICG-001 treated groups (Fig 3).

To evaluate the effect of the Wnt/β-catenin signaling pathway on cell cycle regulation, FACS analysis was performed. Cell cycle and apoptosis analysis kits were used to label cells with PI, which stains DNA quantitatively. The cell percentages of the S, G_2_/M, and G_0_/G_1_ phases were determined by FACS analysis (Fig 3). Results indicated that the percentage of the G_0_/G_1_ phase cells was significantly increased in the AZD2858-treated group and slightly increased in the ICG-001-treated group (Fig 3). By contrast, the percentage of the G_2_/M phase cells was significantly decreased in both AZD2858- and ICG-001-treated groups. The percentage of S phase cells was significantly decreased in the AZD2858-treated group (Fig 3).

**Fig 4:**
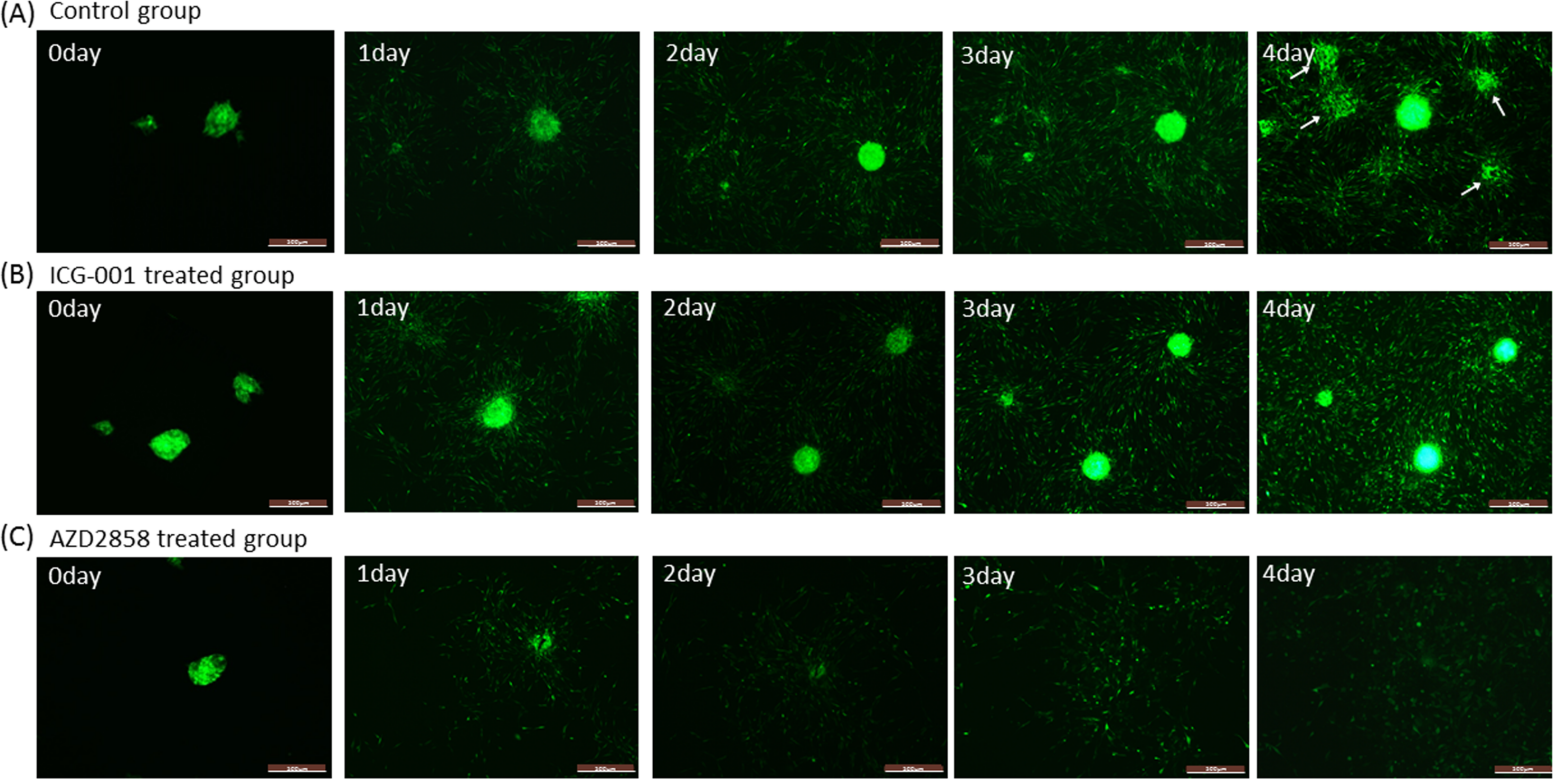
Time-course of EGFP-U87 glioma cell aggregates invasion into Matrigel. 3D tumor spheroid invasion assay. U87 spheroid 3D invasion into Matrigel was monitored every 24 hours using fluorescence microscope. (A) Non-treated EGFP-U87 cells. (B) ICG-001 treated EGFP-U87 cells. (C) AZD2858 treated EGFP-U87 cells. The white arrows indicate newly formed colonies in control group. Bar: 500um.

To evaluate the effects of AZD2858 and ICG-001 on U87 cell invasion, 3D tumor spheroid invasion assay was adopted. Aggregates of EGFP-U87 cells, which were embedded in Matrigel, were equally separated into 3 dishes. AZD2858 and ICG-001 were then added into the cell culture medium used to cover Matrigel. The results showed that the non-treated aggregates in Matrigel spread faster than the AZD2858-treated aggregates (Fig 4). Although the rate of cell spread between the non-treated group and the ICG-001-treated group were difficult to distinguish, we found more newly formed colonies inside the control plates on Day 4 (Fig 4). This result suggested that ICG-001 could not easily inhibit the invasion and migration of U87 aggregates; however, it could inhibit the colony formation by reducing the glioma cell population. By contrast, AZD2858 could significantly inhibit the invasion and migration of U87 glioma cells.

### Gene expression profiles and gene ontology (GO) analysis

**Fig 5:**
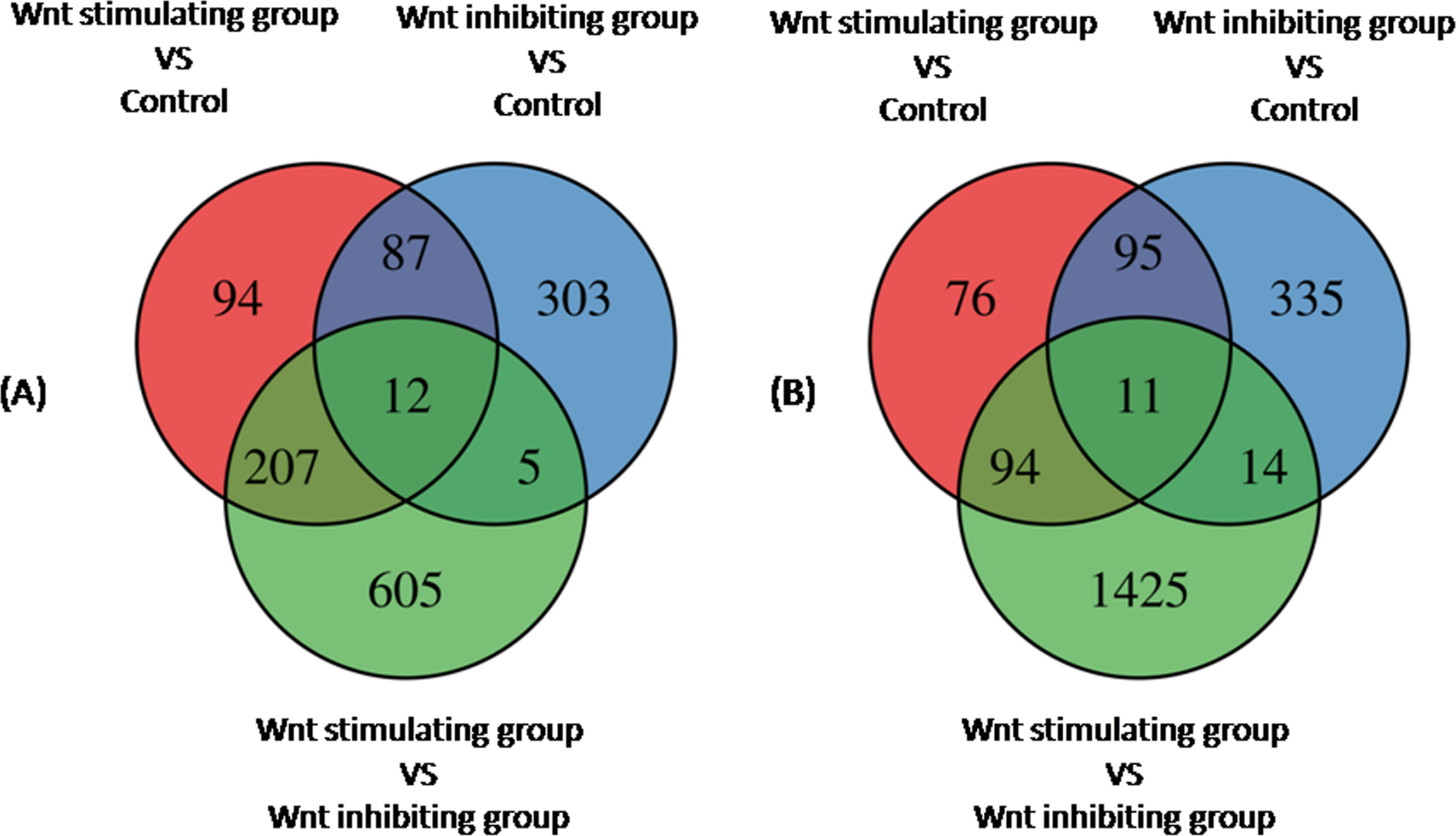
Venn diagrams of differentially expressed genes between different groups. (A) Up-regulated genes between groups. (B) down-regulated genes between groups. The diagram showing the intersection of genes differentially expressed between Wnt stimulating groups and control (red), between Wnt inhibiting group and control (blue), between Wnt stimulating group and Wnt inhibiting group (green). FDR-adjusted p-value<0.05 and fold-change >2.0.

**Fig 6:**
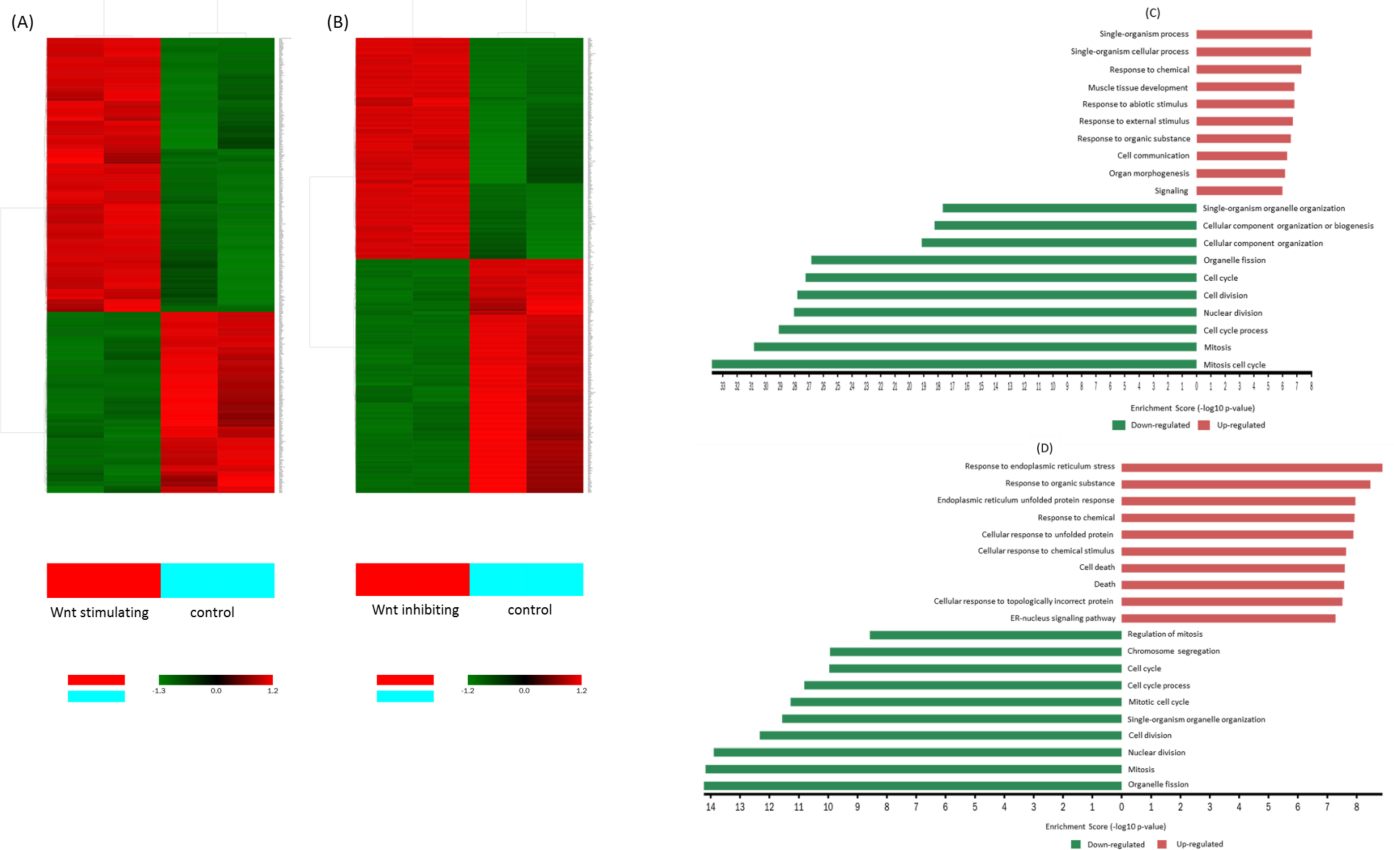
Clustering and characterization of the differential expression of genes. (A) The differential expression genes in Wnt stimulating group, were selected for cluster analysis. (B) The differential expression genes in Wnt inhibiting group, were slected for cluster analysis. (C) Categories of 10 top enriched biological process GO term in Wnt stimulating group. (D) Categories of 10 top enriched biological process GO term in Wnt inhibiting group. FDR-adjusted p-value<0.05 and fold-change >2.0.

To examine the gene profile, Affymetrix GeneChip analyses were performed. Gene Set Enrichment Analysis and Gene Ontology Analysis were used to perform an enrichment analysis on gene sets. The gene expression profiles of Wnt stimulated group and Wnt inhibited group were compared to the non-treated group (control). The relative transcription levels of genes, which showed a fold change more than 2 or less than -2, FDR-adjusted *p*-value less than 0.05 (p<0.05) were considered to be up-regulated/down-regulated. In this study, 400 genes were up-regulated and 276 genes were down-regulated in Wnt stimulation group; 404 genes were up-regulated and 455 genes were down-regulated in Wnt inhibition group (Fig 5). All differential expression genes were annotated on the basis of the gene ontology (GO) database using Visulaization and Integrated Discovery (DAVID). Gene ontology Analysis sorted genes by enriched biological processes (BP) among Wnt stimulated group, Wnt inhibited group and control The 10 most up-regulated and 10 most down-regulated biological processes are listed in S1 Table. The clustering analysis results from three different samples (non-treatment group, Wnt stimulating group, and Wnt inhibiting group) of U87 glioma cells were showed in Fig 6. Interestingly, biological processes such as cell cycle, cell division, nuclear division, cell cycle process, mitosis, and mitosis cell cycle were down-regulated in both AZD2858 treated cells and ICG-001 treated cells. This result supported CCK-8 result, Ki-67 positive cell counting and cell cycle analysis that both inhibiting and stimulating Wnt signaling pathway could inhibit the proliferation of glioma cells. However, the biological processes, which regulates the protein metabolic process, cellular protein modification process, protein phosphorylation and response to stress, were significant down-regulated in AZD2858 treated cells, suggesting that these processes might contribute to more serious inhibition of glioma proliferation via AZD2858 treatment than ICG-001 treatment.

### Pathway analysis

Pathway mapping of differential genes was performed using Kyoto Encyclopedia of Genes and Genomes (KEGG) pathway data base. Significant numbers of differential expression genes from Wnt stimulating/inhibiting groups and control were enriched by KEGG pathway analysis showed that some pathways which involved in cell growth and death, as well as signaling transduction were affected (Table 1). We found that P53 signaling pathway involved in both Wnt activated group and Wnt inhibited group. Meanwhile, cell cycle, ECM-receptor interaction and PI3K-Akt signaling pathway were down-regulated in both groups. These results suggesting that the glioma initiated different strategies to responses to stimulate and inhibit Wnt signaling pathway (Table 2).

**Table 1:** KEGG pathways of differential expression genes involved in cell growth, death and signaling transduction.

Pathway ID	Definition	Count	Fisher-PValue
**Up-regulated KEGG pathways of genes (Wnt stimulated group VS control)**			
hsa04115	p53 signaling pathway	68	3.06322E-05
hsa05217	Basal cell carcinoma	55	4.27718E-05
hsa04390	Hippo signaling pathway	155	7.93574E-05
hsa04010	MAPK signaling pathway	259	0.003566271
hsa04310	Wnt signaling pathway	5	0.005451165
hsa04350	TGF-beta signaling pathway	81	0.0127889
hsa04340	Hedgehog signaling pathway	51	0.03327609
**Up-regulated KEGG pathways of genes (Wnt inhibited group VS control)**			
hsa04142	Lysosome	122	1.12366E-05
hsa04115	p53 signaling pathway	68	0.001574577
hsa04150	mTOR signaling pathway	60	0.01759602
**Up-regulated KEGG pathways of genes (Wnt stimulated group VS Wnt inhibited group)**			
hsa04115	p53 signaling pathway	68	0.004654668
hsa04350	TGF-beta signaling pathway	81	0.01438354
hsa04310	Wnt signaling pathway	143	0.01843059
hsa04390	Hippo signaling pathway	155	0.03313041
hsa04151	PI3K-Akt signaling pathway	344	0.03765752
hsa04010	MAPK signaling pathway	259	0.03962708
**Down-regulated KEGG pathways of genes (Wnt stimulated group VS control)**			
hsa04350	TGF-beta signaling pathway	81	6.54359E-07
hsa04110	Cell cycle	124	6.98491E-07
hsa04512	ECM-receptor interaction	86	7.39772E-05
hsa04115	p53 signaling pathway	68	0.000111613
hsa04390	Hippo signaling pathway	155	0.00020828
hsa04151	PI3K-Akt signaling pathway	344	0.001369607
**Down-regulated KEGG pathways of genes (Wnt inhibited group VS control)**			
hsa04512	ECM-receptor interaction	86	0.000448876
hsa04110	Cell cycle	124	0.005822791
hsa04115	p53 signaling pathway	68	0.009369444
hsa04151	PI3K-Akt signaling pathway	344	0.01031808
**Down-regulated KEGG pathways of genes (Wnt stimulated group VS Wnt inhibited group)**			
hsa04142	Lysosome	122	3.2612E-06
hsa04010	MAPK signaling pathway	259	0.000125015
hsa04210	Apoptosis	88	0.000210252
hsa04151	PI3K-Akt signaling pathway	344	0.0003508434
hsa04115	p53 signaling pathway	68	0.0005903069
hsa04110	Cell cycle	124	0.000671551
hsa04066	HIF-1 signaling pathway	106	0.001046366
hsa04330	Notch signaling pathway	48	0.00160571
hsa04150	mTOR signaling pathway	60	0.004105073
hsa04350	TGF-beta signaling pathway	81	0.004118491

FDR-adjusted p-value<0.05 and fold-change >2.0.

**Table 2:** Differential expression genes analysis base on KEGG in three groups.

Pathway Name	Number		Genes
**Wnt stimulated group VS control**			
p53 signalingpathway Hippo signalingpathway	16 23	CASP3, CCNB1, CCNB2, CCNG1, CDK1, CDKN1A, FAS, GADD45A, GTSE1, PMAIP1, RRM2, RRM2B, SERPINE1, SESN1, SESN2, THBS1, AXIN2, BIRC5, BMP2, BMP4, BMP7, CTGF, DLG1, FZD3, FZD8, ID1, ID2, ID3, ID4, LEF1, NKD1, SERPINE1, SMAD1, SMAD3, SOX2, TCF7, TGFB2, TGFBR1, WNT5A	
TGF-beta signaling pathway	16	BMP2, BMP4, BMP7, DCN, ID1, ID2, ID3, ID4, RBL1, SMAD1, SMAD3, SMAD6, SMAD7, TGFB2, TGFBR1, THBS1	
Cell cycle	16	BUB1, BUB1B, CCNA2, CCNB1, CCNB2, CDC14B, CDC25C, CDK1, CDKN1A, CDKN2D, GADD45A, RBL1, SMAD3, TGFB2, TTK, WEE1	
ECM-receptor interaction	11	CD44, COL3A1, COL4A2, COL4A4, FN1, HMMR, ITGB3, LAMA4, LAMB3, THBS1, TNC	
MAPK signalingpathway	21	CACNA2D3, CASP3, DDIT3, ELK1, FAS, FGF13, FGF20, FGF5, GADD45A, IL1A, IL1R2, MAP3K5, MAP3K8	
Wnt signaling pathway	13	AXIN2, CAMK2D, FZD3, FZD8, LEF1, MMP7, NKD1, PLCB4, RAC1, SMAD3, SOST, TCF7, WNT5A	
PI3K-Akt signaling pathway	21	ANGPT1, BCL2L11, CDKN1A, COL3A1, COL4A2, COL4A4, FGF13, FGF20, FGF5, FN1, ITGB3, LAMA4, LAMB3, PDGFRA, PDPK	
**Wnt inhibited group VS control**			
p53 signaling pathway	13	ATM, CCNB1, CCNB2, CCND1, CCND2, CCNE2, CCNG2, GADD45A, GTSE1, IGFBP3, RRM2B, SESN2, THBS1	
ECM-receptor interaction	12	COL1A1, COL1A2, COL3A1, COL4A2, COL4A6, COL5A2, FN1, ITGA1, ITGB3, ITGB5, LAMA4, THBS1	
PI3K-Akt signaling pathway	31	AKT3, ANGPT1, ATF4, CCND1, CCND2, CCNE2, COL1A1, COL1A2, COL3A1, COL4A2, COL4A6, COL5A2, CSF3, EIF4EBP1, FGF5, FN1, FOXO3, GNG2, GNG4, HGF, ITGA1, ITGB3, ITGB5, LAMA4, NGF, PCK2, PDGFA, PDGFRA, SOS2, THBS1, VEGFA	
Cell cycle	12	ATM, BUB1, BUB1B, CCNB1, CCNB2, CCND1, CCND2, CCNE2, GADD45A, RBL1, SMC1A, TTK	
MAPK signaling pathway	20	AKT3, ATF4, CDC42, DDIT3, DUSP1, FGF5, FOS, GADD45A, HSPA2, IL1A, MAP3K8, MAPK9, MKNK2, NGF, PDGFA, PDGFRA/	
**Wnt stimulated group VS Wnt inhibited group**			
p53 signaling pathway	25	ATM, BAX, CASP3, CASP8, CCND1, CCND2, CCNG1, CDK2, DDB2, FAS, GADD45B, GTSE1, IGFBP3, MDM2, PTEN, RRM2, RRM2B, SERPINE1, SESN2, SHISA5, SIAH1, STEAP3, TNFRSF10B, TP53, ZMAT3	
MAPK signaling pathway	63	AKT1, AKT2, AKT3, ATF2, CACNA1A, CACNA2D3, CACNB2, CASP3, CDC25B, CDC42, CRK, DUSP1, DUSP10, DUSP3, EGFR, ELK1, ELK4, FAS, FGF13, FGF2, FGF20, FGFR1, FLNA, FLNB, FOS, GADD45B, GNA12, HSPA1A, IKBKB, IL1B, IL1R2, MAP2K2, MAP3K3, MAP3K5, MAP3K8, MAPK12, MAPK14, MAPK9, MAX, MEF2C, MKNK2, MYC, NFATC2, NGF, NR4A1, PDGFA, PLA2G4A, PPP3R1, PPP5C, PRKACB, RAC1, RAPGEF2, RPS6KA1, RPS6KA2, RPS6KA5, RRAS, TGFB1, TGFBR1, TGFBR2, TNFRSF1A, TP53, TRAF2, ZAK	
PI3K-Akt signaling pathway	77	AKT1, AKT2, AKT3, ANGPT1, ATF2, BCL2L11, CCND1, CCND2, CDK2, CDKN1B, COL1A2, COL3A1, COL4A4, COL6A2, COL6A3, CREB3L2, CRTC2, CSF3, EGFR, EIF4B, EIF4E2, EIF4EBP1, FGF13, FGF2, FGF20, FGFR1, FN1, FOXO3, GNB2, GNB5, GNG2, GNG4, IKBKB, IL6, IL7R, INSR, ITGA1, ITGA5, ITGB1, ITGB3, ITGB8, JAK2, KITLG, LAMA2, LAMB1, LAMB3, LAMC1, LPAR1, LPAR6, MAP2K2, MDM2, MLST8, MYC, NGF, NR4A1, OSMR, PCK2, PDGFA, PDGFC, PDPK1, PIK3R3, PKN1, PPP2R1A, PPP2R5C, PPP2R5D, PRLR, PTEN, RAC1, RHEB, RXRA, SYK, THBS3, TNC, TP53, VEGFA, VEGFC, YWHAG	
TGF-beta signaling pathway	25	ACVR1, ACVR1B, BAMBI, BMP2, BMP4, BMPR1B, DCN, E2F4, E2F5, ID1, ID2, ID3, INHBA, MYC, PITX2, PPP2R1A, RHOA, SMAD1, SMAD3, SMAD4, SMAD6, SMAD7, TGFB1, TGFBR1, TGFBR2	
Cell cycle	30	ABL1, ATM, CCND1, CCND2, CDC14B, CDC20, CDC25B, CDC45, CDK2, CDKN1B, CDKN2D, E2F4, E2F5, GADD45B, HDAC1, HD	
mTOR signaling pathway	17	AKT1, AKT1S1, AKT2, AKT3, EIF4B, EIF4E2, EIF4EBP1, IKBKB, MLST8, PDPK1, PIK3R3, PTEN, RHEB, RPS6KA1, RPS6KA2, STRADA, VEGFA	
ECM-receptor interaction	22	CD44, COL1A2, COL3A1, COL4A4, COL6A2, COL6A3, DAG1, FN1, ITGA1, ITGA5, ITGB1, ITGB3, ITGB8, LAMA2, LAMB1	
Wnt signaling pathway	32	AXIN2, BAMBI, CAMK2D, CCND1, CCND2, CSNK1E, CSNK2A1, CTNNB1, DKK1, FBXW11, FZD8, LEF1, MAPK9, MMP7, MYC, NFATC2, NKD1, PLCB1, PLCB4, PPP3R1, PRKACB, PSEN1, RAC1, RHOA, SENP2, SIAH1, SMAD3, SMAD4, TCF7, TP53, VANGL1, WNT5A	
HIF-1 signaling pathway	24	AKT1, AKT2, AKT3, ALDOA, ANGPT1, CAMK2D, CDKN1B, EGFR, EGLN2, EIF4E2, EIF4EBP1, HK1, HK2, HMOX1, IFNGR1, IL6, INSR, MAP2K2, MKNK2, PIK3R3, SERPINE1, SLC2A1, TFRC, VEGFA	
VEGF signaling pathway	16	AKT1, AKT2, AKT3, CDC42, MAP2K2, MAPK12, MAPK14, NFATC2, PIK3R3, PLA2G4A, PPP3R1, PXN, RAC1, SPHK1, SRC, VEGFA	
NF-kappa B signaling pathway	20	ATM, BIRC2, BIRC3, CSNK2A1, CXCL2, DDX58, GADD45B, IKBKB, IL1B, IL8, LYN, MALT1, MYD88, PARP1, PLAU, PRKCQ	

FDR-adjusted p-value<0.05 and fold-change >2.0.

## Discussions

### Both inhibiting and stimulating the Wnt/β-catenin signaling pathway reduced the proliferation and survival of U87 glioma cells

The U87 malignant glioma cell line was used in this study. The source of U87 has recently been reported as mislabeled and unknown; however, transcriptional similarity analysis has indicated that five of the five most similar transcriptomes in the U87 cell line are of CNS tumor origin [12]. Therefore, the U87 glioma cell line, when cultured under proper conditions, could still be employed as an experimental glioma model [12]. Previous studies reported that some populations in the U87 cell line exhibit the behaviors and features of cancer stem cells [13–15]. These findings suggested that U87 glioma cells have a heterogeneous cell population, which contains GSCs. Therefore, in this study, U87 cells were used as a high-grade glioma model to evaluate the effects of the Wnt signaling pathway on invasion and proliferation of glioma in vitro.

In this study, glioma specific markers and cancer stem cell markers were checked to identify the glioma origin of U87 cells. The ICC results suggested that the U87 cells used in this study could express a neural progenitor marker NESTIN [16], neuron-specific marker Tuj 1 [17], and glial cell marker GFAP. Additionally, similar to previous reports on GSC characterization [18], the expression of pluripotent stem cell marker OCT4 [19,20],stem cell marker NANOG and SOX2 [18] could be monitored in the U87 cells. The expression of CD133, which can be used as a GSCs marker [14], was also identified in U87 cells. Therefore, the U87 cell line used in this experiment possessed both tumor-specific phenotypes and neural cell phenotypes; therefore, the U87 cell line can be suitably used as a glioma model *in vitro*.

To stimulate and inhibit the Wnt/β-catenin signaling pathway, ICG-001, IWR-1-endo, and AZD2858 were used. ICG-001 antagonizes Wnt/β-catenin/TCF-mediated transcription in a CBP-dependent fashion; thus, it downregulates the transcription of β-catenin/Tcf target genes [21]. ICG-001 also inhibits the proliferation of colorectal cancer cells and decreases the tumor sphere formation of ovarian cancer cells [22]. Furthermore, ICG-001 can inhibit the β-catenin mediated transcriptional axis in stem cells [4,23]. Different from ICG-001, IWR-1-endo inhibits the Wnt/β-catenin pathway by stabilizing the Axin-scaffolded destruction complex [24], and it has been used to treat gliomas in vivo [25]. However, in the current study, IWR-1-endo could significantly reduce cytoplasmic β-catenin but failed to reduce the accumulation of β-catenin in nucleus. Compared with IWR-1-endo, ICG-001 could significantly reduce the accumulation of the nucleic β-catenin in U87 cells. Therefore, in the current study, ICG-001 was used to inhibit the Wnt/β-catenin signaling pathway. AZD2858 is a GSK-3 inhibitor, which activates the transcription of Wnt target genes by recruiting β-catenin into the nucleus where β-catenin forms a transcription complex with TCF/LEF. Western blot analysis suggested that AZD2858 could promote the accumulation of β-catenin in the cytoplasm as well as in the nucleus. Compared with ICG-001, AZD2858 could significantly promote the expression of the target genes of the Wnt/β-catenin signaling pathway. Therefore, AZD2858 and ICG-001 were used as drugs with contrary functions to explore the effects of the Wnt/β-catenin signaling pathway on the behaviors of U87 cells.

Recently, reports showed that the Wnt signaling pathway was associated with the proliferation of glioma cells. We found that treatment of U87 cells with both ICG-001 and IWR-1-endo reduced the proliferation and colony formation of U87 glioma cells. Similar to our finding, Nowicki et al. [26] reported that inhibiting GSK-3 through lithium could reduce the motility of gliomas. Another study reported that inhibiting the expression of Pygo2 could decrease the proliferation, colony formation, and BrdU incorporation of gliomas by reducing cyclin D1, a downstream gene of the Wnt/β-catenin signaling pathway [27]. The conclusion was analogous to the findings by Zhang. They found that interacting β-catenin with FoxM1 could increase the expression of Wnt/β-catenin target genes, thus promoting the cell proliferation and growth of gliomas [28]. In addition, Li et al. [29] indicated that inhibiting miR-92b could promote apoptosis of glioma cells by regulating the expression of Wnt/β-catenin pathway target genes, such as *Dickkopf-3*, a direct target of miR-92b. In addition, knockdown β-catenin by siRNA in U251 glioma cells could inhibit cell proliferation, reduce invasion, and induce apoptosis [8]. Contrary to these findings, we found that activating Wnt/β-catenin in U87 gliomas reduced the proliferation of U87 glioma cells. No evidence has this far shown that increasing the accumulation of nucleic β-catenin in glioma by reducing GSK-3 activation, could suppress cell proliferation, migration, and survival. To compare and contrast the effects of 2 drug treatments on U87 glioma cells, we performed Affymetrix HFU133 Plus 2.0 microarray to analyze the transcriptome of glioma cells.

### The effects of inhibiting and stimulating Wnt/β-catenin signaling pathway on the enrichment of biological processes and signaling pathways

The functional relevance of each gene set was identified using g:GOSt in the g:profiler Web server. First, stimulating and inhibiting the Wnt/β-catenin signaling pathway responded to different biological processes. Some biological processes such as cell cycle, cell division, and mitosis were downregulated in both groups. This result explained the reduction in the proliferation of U87 cells in both groups. However, the cell cycle, cell division, and mitosis, which are involved in cell proliferation, were decreased 33-fold in AZD2858-treated cells compared with 14-fold in ICG-001-treated cells. It may be attributed to the rapid decrease in the cell population in the AZD2858-treated group. However, cell death was markedly increased in ICG-001-treated cells; meanwhile, the enrichment of the single-organism cellular process, single-organism process, and cellular component organization in AZD2858-treated cells was higher than that in ICG-001 treated cells. These findings suggested that the reduced cell population in both groups might dominate by different biological processes.

Second, stimulating and inhibiting the Wnt pathway cross-talk to different pathways. Depending on the fold change value criteria, which indicates the number of deregulated transcripts, some pathways were selected. These pathways were further selected with the adjusted p-value≤ 0.001. As an arbitrary threshold false discovery rate of 0.05 [30], 9 pathways were associated with stimulating the Wnt/β-catenin signaling pathway, and 3 pathways were associated with inhibiting this pathway. Cluster analysis of differentially expressed genes indicated that stimulating and inhibiting the Wnt/β-catenin signaling pathway affected proliferation, survival, and invasion of U87 glioma cells through different mechanisms.

### Stimulating the Wnt/β-catenin signaling pathway with AZD2858 reduced the proliferation, survival, and invasion of the U87 cell

We selected 9 highly enriched pathways. P value was less than 0.001, and FDR value was less than 0.05 [30]. We found that AZD2858 effectively downregulated the TGF-β signaling pathway by decreasing the expression of *TGF-β receptor I*, *Smad2/3*, and *P107*. The TGF-β signaling pathway plays a dual role in tumor suppression and tumor promotion. Katz et al. [31] indicated that the TGF-β signaling pathway regulates the proliferation of various cancers, and it is targeted for glioma treatment. Thacker et al. [32] suggested that downregulating the TGF-β signaling pathway could inhibit the migration and invasion of glioma. In addition, TGF-β inhibitors were used in preclinical and clinical trials as anticancer agents [33]. Therefore, our findings indicated that AZD2858 could be used as an anti-glioma drug to reduce the growth of glioma by inhibiting the TGF-β signaling pathway.

Apart from the TGF-β signaling pathway, the cell cycle pathway was also downregulated with AZD2858 treatment via downregulating the gene expression of *cyclin A2*, *cyclin B3*, and *cyclin dependent kinase 1* (*CDK1*). The protein cyclin A2 regulates the cell cycle by binding to CDK2 and promotes transition via G1/S and G2/M. CDK1 is a subunit of the M-phase promoting factor complex, which controls the transition of the G1/S and G2/M phases. Therefore, in the current study, the downregulation of *cyclin A2*, *cyclin B3*, and *CDK1* genes could prompt cell cycle arrest in U87 glioma cells. Similarly to our findings, Gao et al. [34] indicated that inducing cell cycle arrest by decreasing the expression of SOX9 could suppress the growth of glioma. Thus, inducing cell cycle arrest with AZD2858 treatment may be one of the strategies to reduce glioma growth.

AZD2858 treatment also affects interactions between the ECM components and transmembrane receptors of glioma cells. The expression of *collagen type I alpha 1 chain*, *Laminin submit gamma 3*, and CD44 were significantly downregulated with AZD2858 treatment. Previous studies found that collagen levels were elevated in glioma than in the normal brain, and it significantly affects brain tumor invasion [35,36]. Laminin α2, a marker of aggressive ependymoma, is associated with the growth of glioblastoma stem cells [37]. CD44 is a cell-surface glycoprotein, which participates in the migration of glioma cells [38,39]. Our finding suggested that stimulating the Wnt/β-catenin signaling pathway could reduce the cell invasion of glioma cells by inhibiting the gene expression of *Col1A1*, *LAMC3*, and *CD44*.

In addition, we found that the pathway in cancers was downregulated. The pathway in cancer is made using the KEGG pathway maps for 14 cancers. It contains oncogenes and tumor suppressor genes. In the current study, under the AZD2858 treatment, some transcription factors, such as *Survivin*, *Vascular endothelial growth factor D*, and *ETS proto-oncogen 1* (*ETS1*) were downregulated. Receptors such as *transforming growth factor β receptor 1* (*TGFBR1*), Frizzled class receptor 10 (FZD10), and the platelet-derived growth factor receptor alpha (PDGFRA), were also downregulated. Previous studies suggested that survivin was involved in recurrent malignant glioma [40] and reduced survivin could promote the apoptosis of U87 glioma cells [41]. Wang et al. [41] reported that VEGF-D could act as pro-angiogenic factors; therefore, increased expression of VEGF-D in glioma cells lead to escape mechanisms from the anti-VEGF therapy. Ets-1 also plays a critical role in the proliferation, invasion, and migration of neuroblastoma cells [42]. PDGFRA was also available to control the proliferation of glioma cells via the ERK-dependent mechanism [43]. Therefore, stimulating the Wnt/β-catenin signaling pathway could reduce the invasion, migration, and proliferation of glioma cells by downregulating cancer-associated pathways.

Notably, some signaling pathways were partly upregulated and partly downregulated, such as the Hippo signaling pathway and the P53 signaling pathway. In our study, the Hippo signaling pathway was highly enriched in AZD2858-treated cells. The Hippo signaling pathway is involved in cell survival, proliferation, differentiation, and organ development. This pathway is also one of the tumor suppressor pathways [44,45]. Moreover, it was associated with tumorigenesis. For example, mutation in Lats2, which is one of the components of the Hippo pathway, responded to 40% of mesothelioma cases [46]. Mutation occurred in *NF2*, the hippo signaling pathway upstream gene, causing acoustic neuromas and meningiomas in the brain [47]. We found that the expression of the Hippo signaling pathway target genes, such as *CTGF*, *Birc5*, *Id1*, and *Id2*, were downregulated. This finding suggests that stimulating the Wnt/β-catenin signaling pathway could decrease the population of U87 glioma cells by reducing the expression of anti-apoptotic genes and pro-proliferative genes in the Hippo signaling pathway.

Another important cancer-related pathway, the P53 signaling pathway, was also enriched in AZD2858-treated cells. The P53 signaling pathway was reported to have regulated the Wnt signaling pathway during differentiation of mouse embryonic stem cells [48]. In the present study, we found that *P21*and *growth arrest and DNA-damage-inducible gamma* (*GADD45G*) in P53 cascades, which is involved in G1 arrest and G2 arrest, were significantly upregulated. Furthermore, *ribonucleotide reductase regulatory TP53 inducible subunit M2B* (*RRM2B*), *GADD45G*, and *sestrin 3* (*SESN 3*) in P53 cascades, which are involved in DNA repair and damage prevention, were also upregulated. In addition, the *Fas cell surface death receptor (Fas)*, *caspase 3 (CASP3)*, and *phorbol-12-myristate-13-acetate-induced protein 1 (PMAIP1)*, which are involved in apoptosis, were upregulated via P53 cascades. Therefore, stimulating the Wnt/β-catenin signaling pathway could induce the G1 arrest, G2 arrest, apoptosis, as well as DNA repair and damage prevention in U87 gliomas.

### Both inhibiting and stimulating the Wnt/β-catenin signaling pathway reduced the proliferation and survival of glioma cells

Although the proliferation and survival of U87 glioma cells were significantly inhibited by treatment with both ICG-001 and AZD2858, these inhibitors played diverse roles in regulating cell behavior. The lysosome pathway was only upregulated in the ICG-001-treated cells. Inhibiting the Wnt/β-catenin signaling pathway by ICG-001 could increase the gene expression of certain lysosomal acid hydrolases, such as *cathepsin C* (*CTSC*), *tripeptidy1 peptidase 1* (*TPP1*), *glucosylceramidase β (GBA)*, *glucosidase alpha (GAA)*, *N-acetyl-alpha-glucosaminidase (NAGLU)*, *hexosaminidase subunit alpha (HEXA)*, *mannosidase β (MANBA)*. The gene expression of *deoxyribonuclease 2*, which encodes nuclease, was also upregulated. Moreover, the gene expression of *N-acylsphingosine amidohydrolase 1* (*ASAH1*), which encodes ceramidase, was upregulated. Other lysosomal enzymes and activators, such as *prosaposin* and the *GM2 ganglioside activator (GM2A)*w, were also upregulated. Lysosomal acid hydrolases are hydrolytic enzymes within lysosomes. They contain degradative enzymes, such as nucleases, proteases, glycosidases, lipases, phosphatases, and sulfatases. The significantly upregulated lysosomal acid hydrolase genes suggest that the function of lysosomes might be activated under the treatment of ICG-001.

We found that the ECM-receptor interaction pathway and the focal adhesion pathway were downregulated in ICG-001 treated cells. These two pathways were also downregulated in AZD2858-treated cells. However, treatment with AZD2858 and ICG-001 affected the expression of distinct genes in these pathways. Except for *COL1A1* and *LAMC3*, *fibronectin 1* was also downregulated in ICG-001-treated cells. FN1 encodes fibronectin, which is an extracellular matrix protein. Fibronectin is involved in cell adhesion and cell migration; it significantly influences embryogenesis, wound healing, and metastasis. It also expressed on the surfaces of glial cells in the brain [49]. Moreover, it is localized in the capillary and meningeal structures of the adult rat brain [50]. No evidence has thus far shown that fibronectin is associated with glioma.

## Conclusions

We found that both the inhibiting and stimulating Wnt/β-catenin signaling pathway could reduce the proliferation of U87 glioma cells. The stimulating Wnt/β-catenin signaling pathway could efficiently inhibit the invasion and survival of U87 glioma cells by GSK-3 inhibition. Interestingly, AZD2848 and ICG-001 treatment caused decreases in the cell population via distinct pathways. These pathways, which cross-talk with Wnt/β-catenin signaling pathway, involved the proliferation, differentiation, and apoptosis of U87 cells.

## Supporting information

S1 Table Top 10 biological process GO terms of differential expression genes resulted from pairwise comparison among three groups. FDR-adjusted p-value<0.05 and fold-change >2.0.
(DOCX)
