# Supplementary material for: Wnt/β-catenin signaling pathway inhibits the proliferation and apoptosis of U87 glioma cells via different mechanisms
Source: PLoS One. 2017 Aug 24;12(8):e0181346. doi: 10.1371/journal.pone.0181346 (PMC5570310; doi:10.1371/journal.pone.0181346)
Supplement: S1 Table — FDR-adjusted p-value<0.05 and fold-change >2.0. (DOCX) [file pone.0181346.s001.docx]

**S1 Table. Top 10 biological process GO terms of differential expression genes resulted from pairwise comparison among three groups.**

| **GO ID** | **Terms** | **Count** | ***P*-value** | **FDR** |
| --- | --- | --- | --- | --- |
| Up-regulated biological process GO terms (Control VS Wnt stimulated group) | | | | |
| GO:0044699 | single-organism process | \| 303 \| \| --- \| | 8.96042E-09 | 2.87089E-05 |
| GO:0044763 | single-organism cellular process | 281 | 1.10888E-08 | 2.87089E-05 |
| GO:0042221 | response to chemical | 111 | 4.86001E-08 | 8.38837E-05 |
| GO:0060537 | muscle tissue development | 24 | 1.49168E-07 | 0.000155252 |
| GO:0009628 | response to abiotic stimulus | 46 | 1.49915E-07 | 0.000155252 |
| GO:0009605 | response to external stimulus | 74 | 1.91446E-07 | 0.000165218 |
| GO:0010033 | response to organic substance | 84 | 2.63966E-07 | 0.000195259 |
| GO:0007154 | cell communication | 158 | 4.80168E-07 | 0.000310789 |
| GO:0009887 | organ morphogenesis | 41 | 6.70928E-07 | 0.000386007 |
| GO:0023052 | signaling | 155 | 1.01699E-06 | 0.000478724 |
| Up-regulated biological process GO terms (Control VS Wnt inhibited group) | | | | |
| GO:0034976 | response to endoplasmic reticulum stress | 18 | 1.31966E-09 | 6.83319E-06 |
| GO:0010033 | response to organic substance | 92 | 3.38841E-09 | 8.77259E-06 |
| GO:0030968 | endoplasmic reticulum unfolded protein response | 14 | 1.11265E-08 | 1.33948E-05 |
| GO:0042221 | response to chemical | 116 | 1.17921E-08 | 1.33948E-05 |
| GO:0034620 | cellular response to unfolded protein | 14 | 1.29343E-08 | 1.33948E-05 |
| GO:0070887 | cellular response to chemical stimulus | 85 | 2.30935E-08 | 1.72272E-05 |
| GO:0008219 | cell death | 79 | 2.53948E-08 | 1.72272E-05 |
| GO:0016265 | death | 79 | 2.66159E-08 | 1.72272E-05 |
| GO:0035967 | cellular response to topologically incorrect protein | 14 | 3.06124E-08 | 1.76123E-05 |
| GO:0006984 | ER-nucleus signaling pathway | 14 | 5.24146E-08 | 2.71403E-05 |
| Up-regulated biological process GO terms (Wnt stimulated group VS Wnt inhibited group) | | | | |
| GO:0009653 | anatomical structure morphogenesis | 145 | 6.3644E-07 | 0.002434199 |
| GO:0048646 | anatomical structure formation involved in morphogenesis | 72 | 1.20812E-06 | 0.002434199 |
| GO:0072358 | cardiovascular system development | 67 | 1.88042E-06 | 0.002434199 |
| GO:0072359 | circulatory system development | 67 | 1.88042E-06 | 0.002434199 |
| GO:0048856 | anatomical structure development | 248 | 3.7648E-06 | 0.003898826 |
| GO:0040011 | locomotion | 95 | 1.18902E-05 | 0.00765348 |
| GO:0070972 | protein localization to endoplasmic reticulum | 18 | 1.21171E-05 | 0.00765348 |
| GO:0001568 | blood vessel development | 46 | 1.33559E-05 | 0.00765348 |
| GO:0048731 | system development | 214 | 1.43531E-05 | 0.00765348 |
| GO:0006928 | cellular component movement | 104 | 1.47808E-05 | 0.00765348 |
| Down-regulated biological process GO terms (Control VS Wnt stimulated group) | | | | |
| GO:0000278 | mitotic cell cycle | 74 | 1.73912E-34 | 9.00517E-31 |
| GO:0007067 | mitosis | 49 | 1.50877E-31 | 3.90622E-28 |
| GO:0022402 | cell cycle process | 78 | 8.06605E-30 | 1.3922E-26 |
| GO:0000280 | nuclear division | 53 | 8.90383E-29 | 1.1526E-25 |
| GO:0051301 | cell division | 62 | 1.52207E-28 | 1.57625E-25 |
| GO:0007049 | cell cycle | 87 | 5.72394E-28 | 4.93976E-25 |
| GO:0048285 | organelle fission | 53 | 1.43605E-27 | 1.06227E-24 |
| GO:0016043 | cellular component organization | 144 | 7.29522E-20 | 4.72183E-17 |
| GO:0071840 | cellular component organization or biogenesis | 144 | 5.72305E-19 | 3.29266E-16 |
| GO:1902589 | single-organism organelle organization | 77 | 2.15834E-18 | 1.11759E-15 |
| Down-regulated biological process GO terms (Control VS Wnt inhibited group) | | | | |
| GO:0048285 | organelle fission | 49 | 5.85548E-15 | 1.72907E-11 |
| GO:0007067 | mitosis | 40 | 6.67854E-15 | 1.72907E-11 |
| GO:0000280 | nuclear division | 47 | 1.27067E-14 | 2.19318E-11 |
| GO:0051301 | cell division | 55 | 4.69534E-13 | 6.07811E-10 |
| GO:1902589 | single-organism organelle organization | 91 | 2.74117E-12 | 2.83875E-09 |
| GO:0000278 | mitotic cell cycle | 59 | 5.18543E-12 | 4.47503E-09 |
| GO:0022402 | cell cycle process | 69 | 1.52579E-11 | 1.12865E-08 |
| GO:0007049 | cell cycle | 81 | 1.08638E-10 | 6.61501E-08 |
| GO:0007059 | chromosome segregation | 22 | 1.14977E-10 | 6.61501E-08 |
| GO:0007088 | regulation of mitosis | 17 | 2.63321E-09 | 1.36347E-06 |
| Down-regulated biological process GO terms (Wnt stimulated group VS Wnt inhibited group) | | | | |
| GO:0044267 | cellular protein metabolic process | 460 | 1.37465E-22 | 7.11792E-19 |
| GO:0006464 | cellular protein modification process | 371 | 2.28315E-21 | 3.94072E-18 |
| GO:0036211 | protein modification process | 371 | 2.28315E-21 | 3.94072E-18 |
| GO:0051246 | regulation of protein metabolic process | 257 | 4.16525E-21 | 5.39191E-18 |
| GO:0032268 | regulation of cellular protein metabolic process | 233 | 6.99896E-21 | 7.24812E-18 |
| GO:0043412 | macromolecule modification | 379 | 1.36579E-20 | 1.17868E-17 |
| GO:0019538 | protein metabolic process | 524 | 2.23985E-20 | 1.65685E-17 |
| GO:0006468 | protein phosphorylation | 200 | 4.98476E-19 | 3.22639E-16 |
| GO:0016310 | phosphorylation | 237 | 1.42828E-18 | 8.21734E-16 |
| GO:0006950 | response to stress | 419 | 2.22864E-18 | 1.15399E-15 |

FDR-adjusted p-value<0.05 and fold-change >2.0.
